# Pentosan Polysulfate Maculopathy With 13 Years of Follow-up Imaging

**DOI:** 10.1177/24741264241228375

**Published:** 2024-01-31

**Authors:** Ashlyn M. Pinto, Nieraj Jain, R. Rishi Gupta

**Affiliations:** 1 Department of Ophthalmology & Visual Sciences, Dalhousie University, Halifax, NS, Canada; 2 Department of Ophthalmology, Emory University School of Medicine, Atlanta, GA, USA

**Keywords:** pentosan polysulfate maculopathy, interstitial cystitis, pigmentary retinopathy, Elmiron, toxicity

## Abstract

**Purpose:** To describe a case of pentosan polysulfate maculopathy progression with 13 years of follow-up imaging. **Methods:** A case was analyzed and a literature review performed. **Results:** A 65-year-old woman was referred to the retina service for a second opinion of a bilateral progressive pigmentary maculopathy. Her medical history was significant for interstitial cystitis that was actively treated with daily pentosan polysulfate since 2003. Multimodal imaging and fundus examination were consistent with pentosan polysulfate maculopathy. A review of records showed previous fundus imaging dating back 13 years that permitted longitudinal assessment of the disease course. Imaging findings were more prominent than the fundus examination findings. There was a 5-year period from the onset of parafoveal atrophy to foveal involvement. A pseudopodial pattern of disease expansion was seen on fundus autofluorescence. **Conclusions:** To our knowledge, this case represents the longest documented follow-up imaging of the progression of pentosan polysulfate maculopathy in the literature.

## Introduction

Interstitial cystitis is a noninfectious syndrome that affects the bladder and is characterized by chronic pelvic pain and urinary urgency.^1^ The pathophysiology is poorly understood and thought to be multifactorial, with theories surrounding autoimmune urothelial dysfunction, mast cell dysfunction, and neurogenic inflammation.^1^ Although treatment for interstitial cystitis can be conservative and includes physical therapy and stress avoidance, pentosan polysulfate sodium remains a popular first-line or second-line pharmacologic treatment in many international guidelines.^1,2^ Pentosan polysulfate has been widely prescribed for interstitial cystitis since its approval by the US Food and Drug Administration in 1996 under the brand name Elmiron (Janssen Pharmaceuticals, Inc).^3^

The estimated prevalence of people with interstitial cystitis in the United States is at least 1 million. Given that, Lindeke-Myers et al^2^ estimate that thousands of patients are at risk for pentosan polysulfate maculopathy. Because pentosan polysulfate maculopathy was first described in 2018, many patients have likely been misdiagnosed with other macular diseases, such as macular degeneration or pattern dystrophies.^4^

We present a case of a 65-year-old woman who had taken pentosan polysulfate for a total of 19 years and was followed for 13 years with a progressive bilateral pigmentary maculopathy of unknown origin. A fundus examination and multimodal imaging of this patient’s condition were in keeping with pentosan polysulfate maculopathy. To our knowledge, this case represents the longest follow-up imaging in a patient continuing pentosan polysulfate treatment in the literature.

## Case Report

A 65-year-old woman presented to the retina service for a second opinion of a bilateral progressive pigmentary maculopathy being followed for 13 years by retina colleagues, who categorized it as a form of macular degeneration under the umbrella of retinal pigment epithelium (RPE) dystrophies. She reported a painless decrease in visual acuity (VA) in both eyes, described as looking through a “honeycomb” pattern with a purple–blue tint.

The patient’s medical history was significant for interstitial cystitis, Sjögren syndrome, hypothyroidism, asthma, arthritis, colitis, and anxiety. Her ocular history included cataract surgery and keratorefractive surgery in both eyes. The patient’s medications included pentosan polysulfate, levothyroxine, imipramine, pregabalin, and salbutamol. Specifically, she had taken pentosan polysulfate for 19 years at a dosage of 200 mg orally per day. The family history was significant for glaucoma and age-related macular degeneration. The social history was significant for modest alcohol intake and use of cannabidiol oil. She had a remote history of smoking 5 cigarettes a day periodically over 30 years, which she had quit 3 years previously.

On examination, the corrected VA was 20/100 OD and 20/50^−1^ OS, with no pinhole improvement. There was no relative afferent pupillary defect, and the intraocular pressure was 8 mm Hg and 7 mm Hg in the right eye and left eye, respectively. A slitlamp examination of both eyes was unremarkable, with posterior chamber intraocular lenses in place. A funduscopic evaluation showed normal nerves with bilateral pigmentary changes and focal atrophy in the macula.

**Figure 1.**
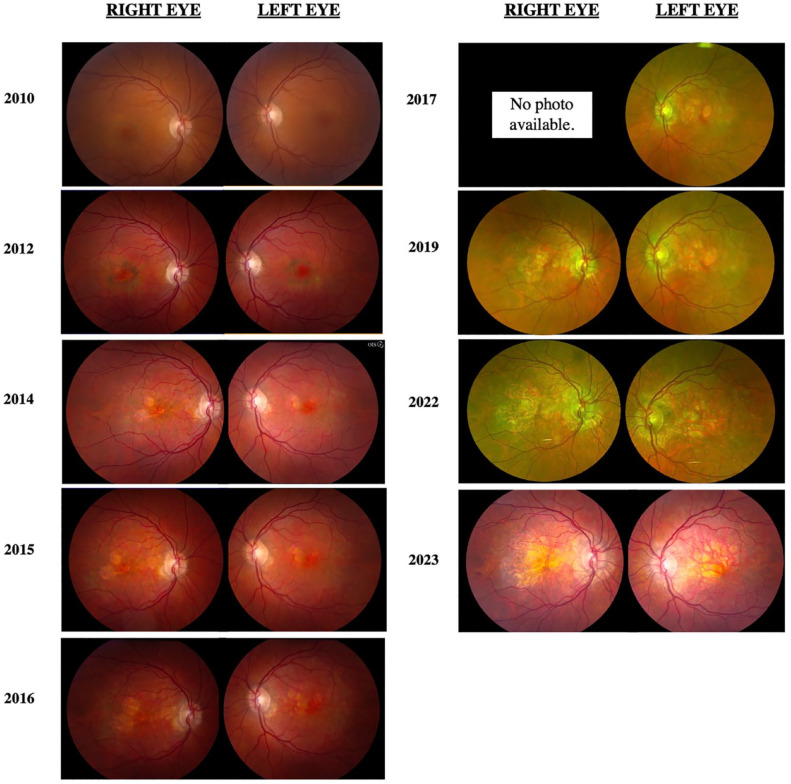
Progression of color and pseudo-color fundus photographs. Color imaging initially shows bilateral macular pigmentary changes that later lose pigment and become focal atrophic areas.

**Figure 2.**
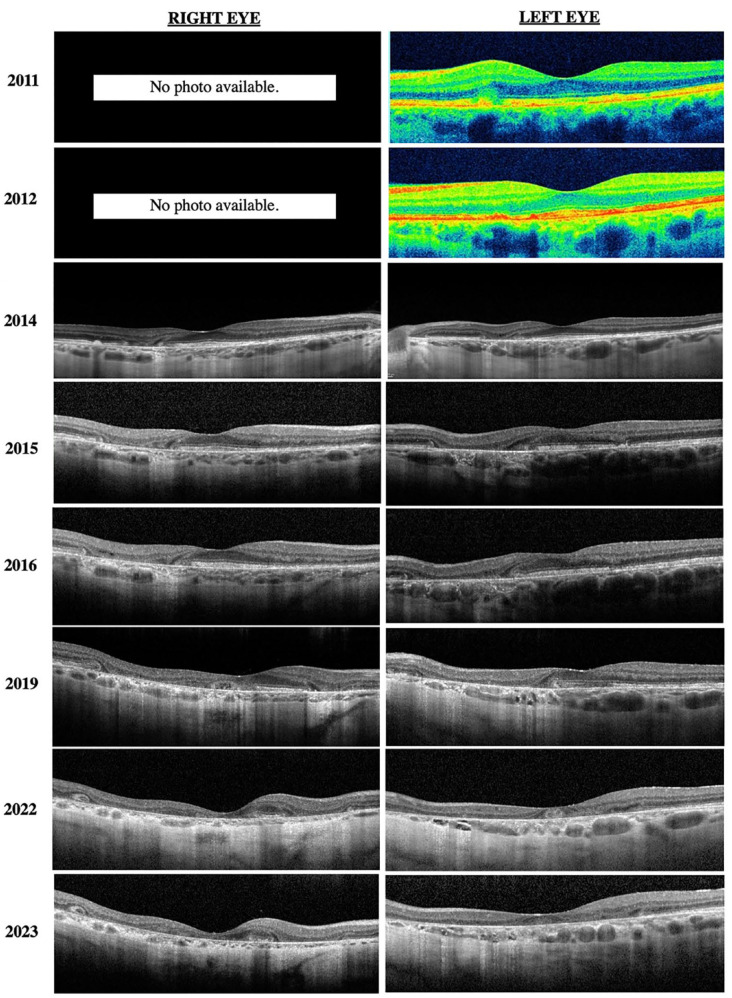
Progression of OCT of both eyes. Initial time-domain OCTs from 2011 and 2012 of the left eye show hyperreflective nodules at the level of the retinal pigment epithelium. Progression of the OCTs shows loss of the normal foveal contour, retinal atrophy, loss of the ellipsoid zone, and outer retinal tubulations. Abbreviation: OCT, optical coherence tomography.

**Figure 3.**
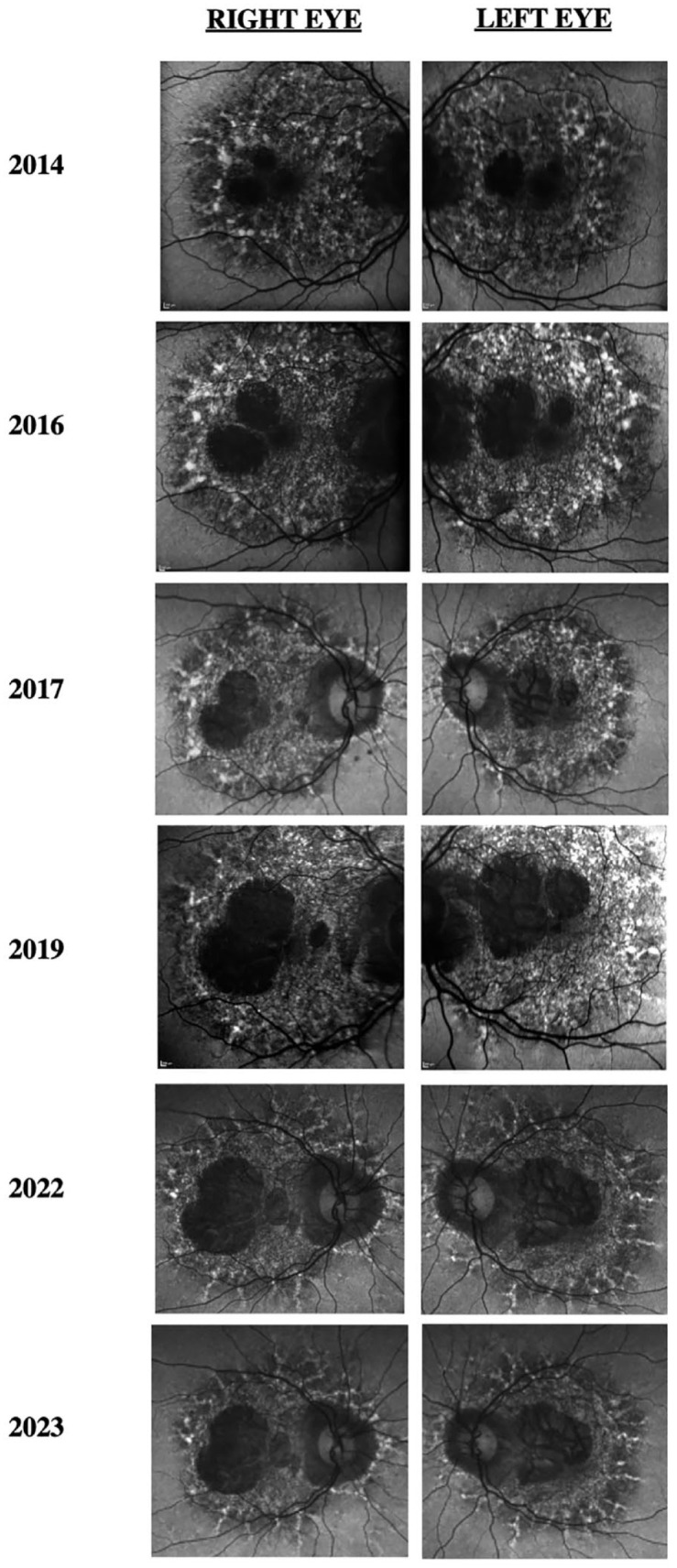
Progression of fundus autofluorescence images of both eyes shows increased peripapillary and macular hypoautofluorescence with surrounding hyperautofluorescent flecks expanding outward from the macula in a pseudopodial pattern.

**Figure 4.**
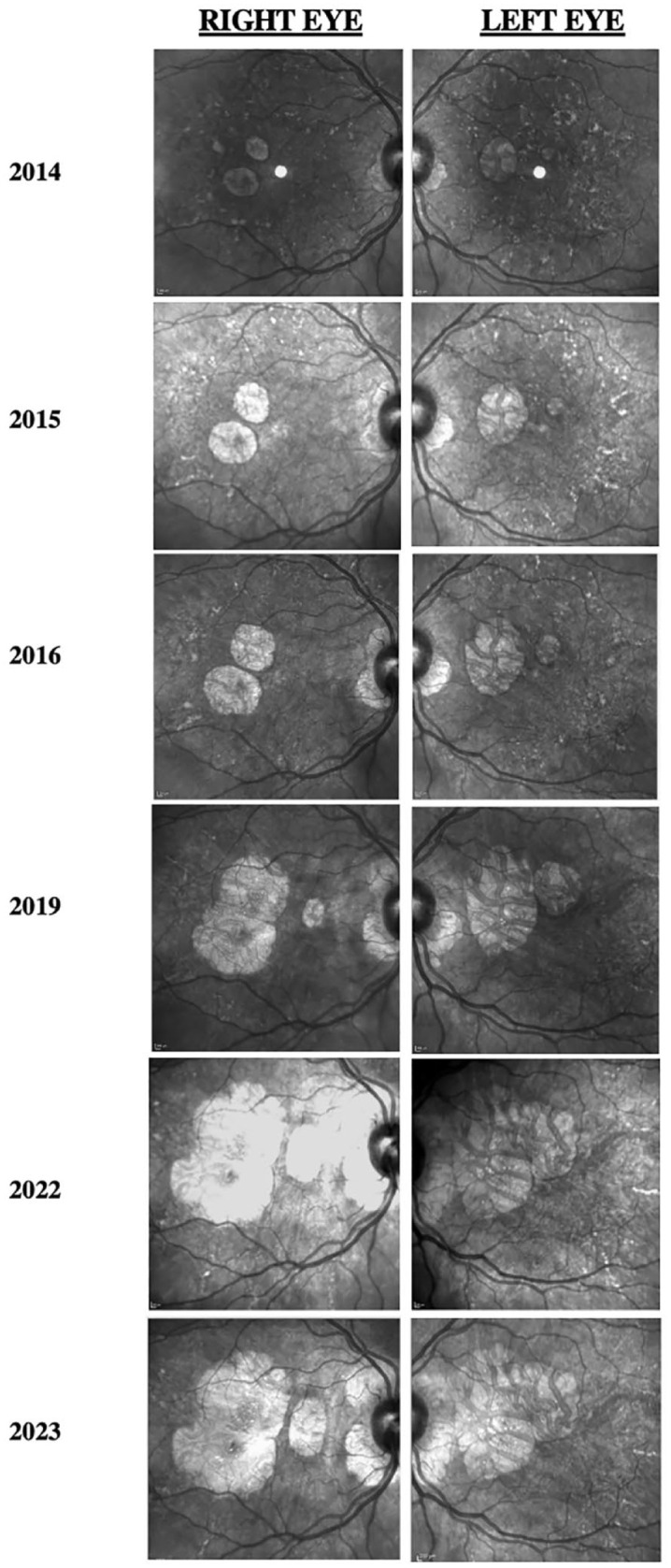
Progression of near-infrared reflectance images of both eyes. Infrared imaging shows a loss over time of hyperreflective flecks in the macula and increased atrophic areas.

**Figure 5.**
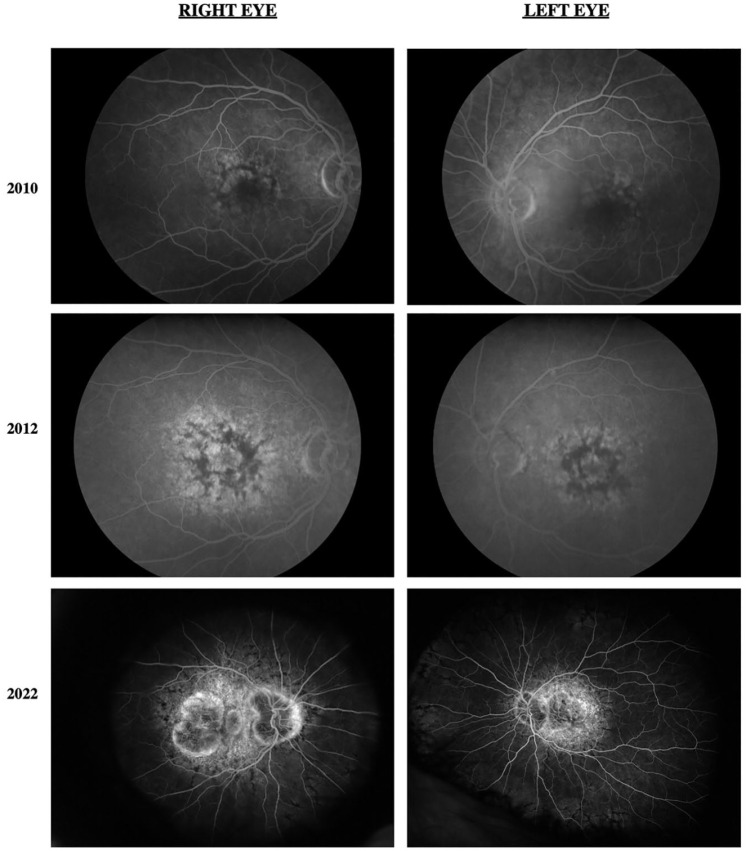
Fluorescein angiography of both eyes initially shows hypofluorescence in the macula with hyperfluorescent spots. Progressive images show expanding macular hyperfluorescence and peripapillary hypofluorescence surrounded by hyperfluorescence.

**Figure 6.**
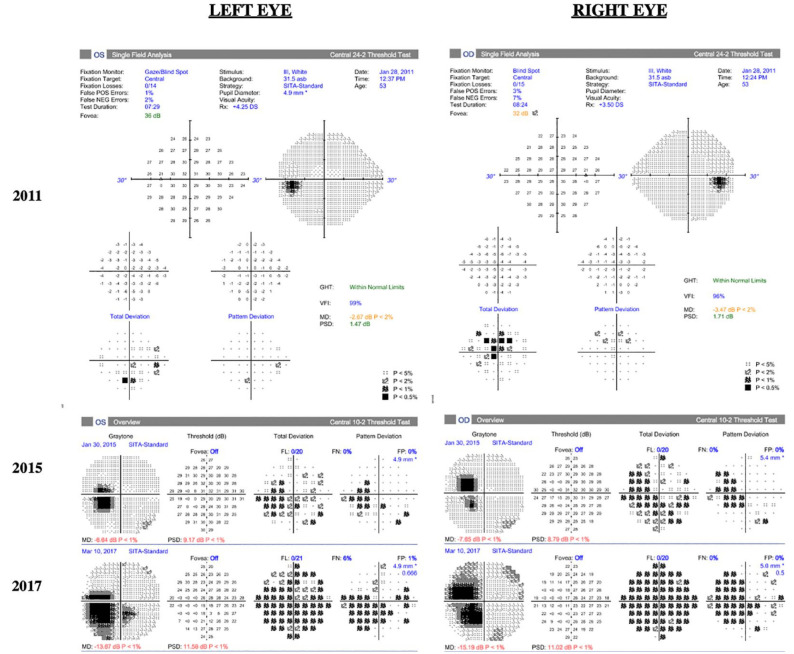
Progression of Humphrey visual fields. A 24-2 Humphrey visual field test of the left eye and right eye performed within the first year of presentation (2011) after 8 years of pentosan polysulfate treatment. The left eye has nonspecific visual defects, and the right eye has a central scotoma. Humphrey visual fields of the left eye and right eye in 2015 and 2017 are 10-2 and show worsening central scotomas bilaterally after 12 years and 14 years on pentosan polysulfate treatment, respectively.

Figure 1 shows the progression of fundus photographs over time. Spectral domain-optical coherence tomography showed outer retinal atrophy. Figure 2 shows the progression of OCTs over time. Outer retinal tubulations were also visible on OCT. Fundus autofluorescence (FAF) imaging showed a peripapillary ring of hypoautofluorescence as well as fovea-involving hypoauotofluorescence that correlated with the atrophy noted on the funduscopic examination (Figure 3). Surrounding these hypoautofluorescent patches were flecks of hyperautofluorescence in the macular and peripapillary regions. Figure 4 shows the patient’s previous near-infrared reflectance imaging over time. Figure 5 shows the patient’s previous fluorescein angiography imaging and Figure 6, the patient’s available formal visual fields over time.

Electrophysiology studies had been performed in the past. In 2010, a multifocal electroretinogram (mfERG) showed supranormal amplitude foveal responses with slightly delayed implicit time. In 2012, the mfERG was similar, with foveal responses of unusually high amplitude for an adult of her age (55 years at the time of testing). In 2014, a full-field ERG showed response amplitudes that were in the lower range of normal in scotopic and photopic conditions, with the mfERG still documenting high-amplitude foveal activity and consistent implicit time delay. In 2014, normal Arden ratios were seen on electrooculography. The full-field ERG from 2023 was within normal limits, and the mfERG showed decreased amplitudes centrally and paracentrally, delayed implicit timing paracentrally, and normal pericentral amplitudes.

## Conclusions

Pigmentary maculopathy secondary to pentosan polysulfate was first described in 2018 by Pearce et al^4^ in a retrospective case series of 6 patients reporting difficulty reading and prolonged dark adaptation after chronic pentosan polysulfate exposure. Initial fundus findings included parafoveal pigmentary changes with subsequent atrophy in several eyes, with the extent of the disease more apparent on multimodal imaging than on funduscopic examination.

A recent review article by Lindeke-Myers et al^2^ described the key clinical features of this toxic form of retinopathy. Similar to our case, the mean (±SD) age at the diagnosis of pentosan polysulfate maculopathy was 62.2 ± 13.2 years. The mean duration of pentosan polysulfate use in those patients with maculopathy was 15.0 ± 5.7 years, with a mean cumulative exposure of 1824 ± 1042 g.^2^ Comparably, our patient was on pentosan polysulfate treatment for 19 years, with a mean cumulative exposure of approximately 1387 g. Vora et al^5^ reported a dose-response relationship, with patients having more than 1500 g of cumulative exposure almost 5 times as likely to have pentosan polysulfate maculopathy as those with 500 to 999 g of cumulative exposure. The median VA of cases is documented at 20/25, with significant loss of acuity typically resulting from atrophic lesions affecting the fovea.^2^

Our patient initially presented in 2010 with 20/25 VA bilaterally. Secondary to foveal atrophy, her most recent VA in 2022 was documented as 20/100 OD and 20/50 OS. Fundus photography typically shows hyperpigmented macular spots, deep yellow-appearing subretinal deposits and, in advanced cases, paracentral RPE atrophy.^2^ Our patient had evident parafoveal pigment clumps in 2012 that ultimately gave way to patchy parafoveal atrophy in subsequent years. Our case had a 5-year period from initial observation of parafoveal atrophy to involvement of the foveal center. OCT of pentosan polysulfate maculopathy showed hyperreflective nodules at the RPE that cast shadows on the underlying choroid, retinal, and choroidal thinning in addition to RPE atrophy.^2^ These hyperreflective nodules were seen on time-domain OCTs earlier in the patient’s course of treatment, with atrophy and outer retinal tubulations developing in subsequent years.

Hadad et al^6^ found a correlation between the ratio of foveal to parafoveal thickness and cumulative pentosan polysulfate exposure in 17 patients. Further research that includes more patients with earlier stage disease would be beneficial if OCT allows for early detection of pentosan polysulfate maculopathy. Over time, our patient’s choroid also appeared on OCT to progressively thin.

FAF imaging typically shows disproportionately more abnormalities than fundus photography, with symmetric, densely packed hyperautofluorescent and hypoautofluorescent spots in the macula. A unique finding of pentosan polysulfate maculopathy vs hereditary maculopathies is the presence of a hypoautofluorescent peripapillary halo on FAF,^2,7^ which occurred in our case. Furthermore, FAF showed that our patient had slow expansion of disease over 10 years. In 2014, the pentosan polysulfate maculopathy was confined in the arcades. In 2023, the maculopathy had slightly expanded past the arcades in a pseudopodial pattern that has not been described previously. On OCT, near-infrared reflectance imaging also showed hyperreflectivity within the macula correlating to RPE hyperreflective nodules.

Furthermore, electrophysiology results in the literature have shown full-field ERGs with mild attenuation of response amplitudes and a delay of cone-derived and rod-derived responses, and mfERGs have shown attenuation of response amplitudes. Electrooculographic testing in cases has found a normal Arden ratio,^8^ as seen in our patient.

Last, although our patient did not present with cystoid macular edema (CME) or macular neovascularization (MNV), both have been reported in previous series.^2^ In these series, the MNV responded successfully to antivascular endothelial growth factor injections.

The pathophysiology of pentosan polysulfate maculopathy is not well understood. Pentosan polysulfate has a structure similar to that of glycosaminoglycans and has been found to have anticoagulant, anti-inflammatory, and fibrinolytic properties.^9^ One theory suggests that its sulfate groups carry a high negative charge and interact nonspecifically with positively charged compounds.^2^ Ancillary imaging of pentosan polysulfate maculopathy does primarily point to disease at the level of the RPE and photoreceptor interface. Because pentosan polysulfate is similar in structure to glycosaminoglycans, hypotheses have suggested that pentosan polysulfate disturbs the glyscosaminoglycan-full matrix between photoreceptors that structurally supports the RPE–photoreceptor interface.^2,10,11^ A recent study with OCT angiography found that choriocapillaris flow impairment may predate imaging findings of RPE disease.^12^ Pentosan polysulfate has also been shown to inhibit fibroblast growth factor in vitro. Greenlee et al^13^ proposed that chronic inhibition of this fibroblast growth factor, which is important for maintaining a healthy retina, leads to retinal damage.^13,14^

In 2019, Health Canada released a product monograph update on pentosan polysulfate regarding the risk for pigmentary maculopathy.^15^ Screening recommendations in a study sponsored by The Macula Society suggest annual ophthalmic evaluation with multimodal imaging, including OCT, FAF or, when available, near-infrared reflectance.^16^ After confirmation of pentosan polysulfate maculopathy, a discussion on drug cessation, or tapering if patients are unable to discontinue the drug because of its therapeutic benefits, is recommended.^2,15^ Unfortunately, some patients may still experience progression of pentosan polysulfate maculopathy despite cessation of pentosan polysulfate treatment.^17^ Although sequelae of pentosan polysulfate maculopathy, such as CME or MNV, can be treated, there is no current treatment for the underlying maculopathy.

Advancements in multimodal imaging have allowed clinicians to characterize abnormalities of the retina in striking detail. Because pentosan polysulfate maculopathy is a relatively newly described entity, further analyses of patients taking pentosan polysulfate long term will help create screening guidelines for this medication, which is associated with potentially avoidable permanent visual deterioration.
